# Supplementary material for: Effects of Acute Hyperthermia on the Thermotolerance of Cow and Sheep Skin-Derived Fibroblasts
Source: Animals (Basel). 2020 Mar 25;10(4):545. doi: 10.3390/ani10040545 (PMC7222367; doi:10.3390/ani10040545)
Supplement: Supplementary file 1 [file animals-10-00545-s001.zip › Supplementary Figures- clean.docx]

**
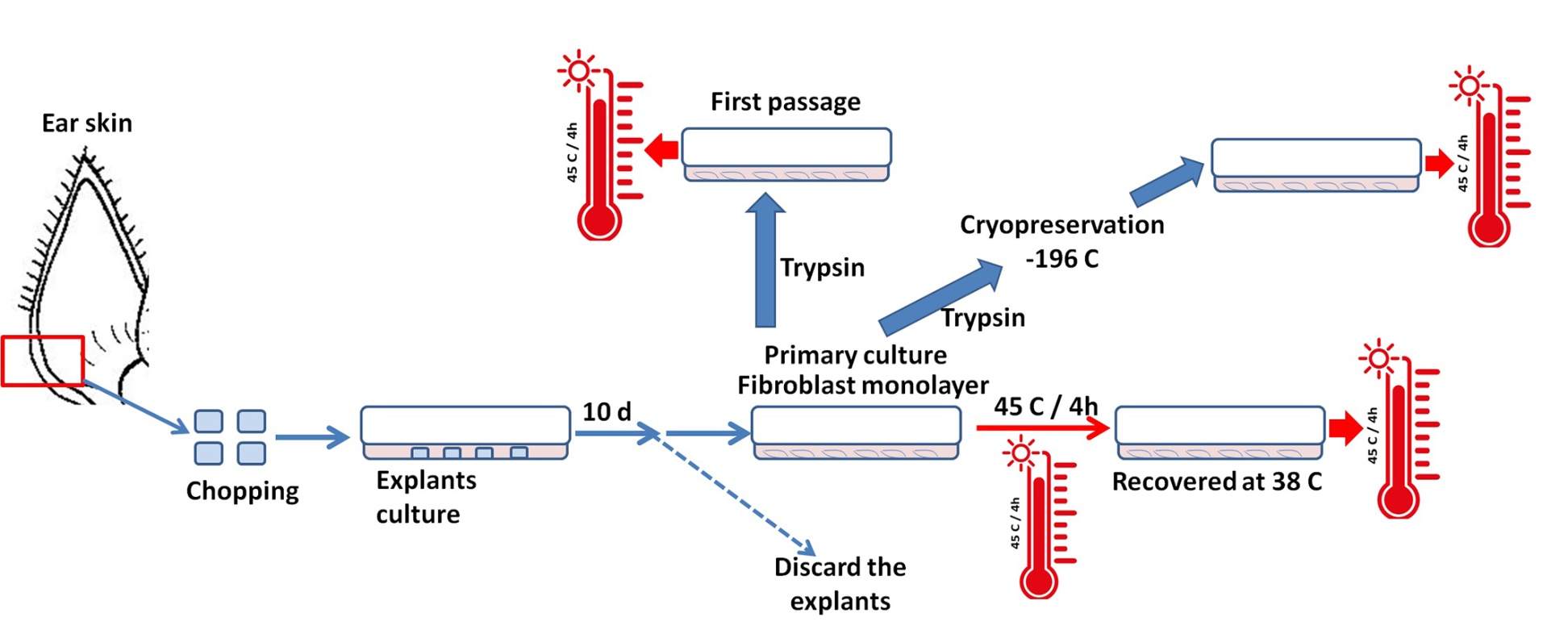
**

**Supplemental Fig. S1. Experimental design flowchart.** Ear skin samples were aseptically prepared as mentioned in the Material and Methods, chopped into pieces of around 1 mm^3^, and cultured in cell culture plates. After attachment and observing fibroblast outgrowths on day 10, the explants were discarded and the fibroblasts were maintained in the culture plates until they reached 70%–80% confluence (primary fibroplast culture). This primary culture was exposed to three different treatments: 1) acute heat shock at 45 °C for 4 h, which was examined for several parameters, such as wound-healing and viability assays; 2) some heat-shocked cells were left for recovery at 38 °C until they reached the initial spindle morphology and 70%–80 % confluence (recovered cells), and then reheated again at 45 °C for 4 h and examined as before; 3) cells were subcultured with the aid of trypsin-EDTA and the resulting first passage was thermally treated as mentioned, and 4) cells were trypsinized and cryopreserved in liquid nitrogen, the thawed and thermally treated as mentioned above.

**
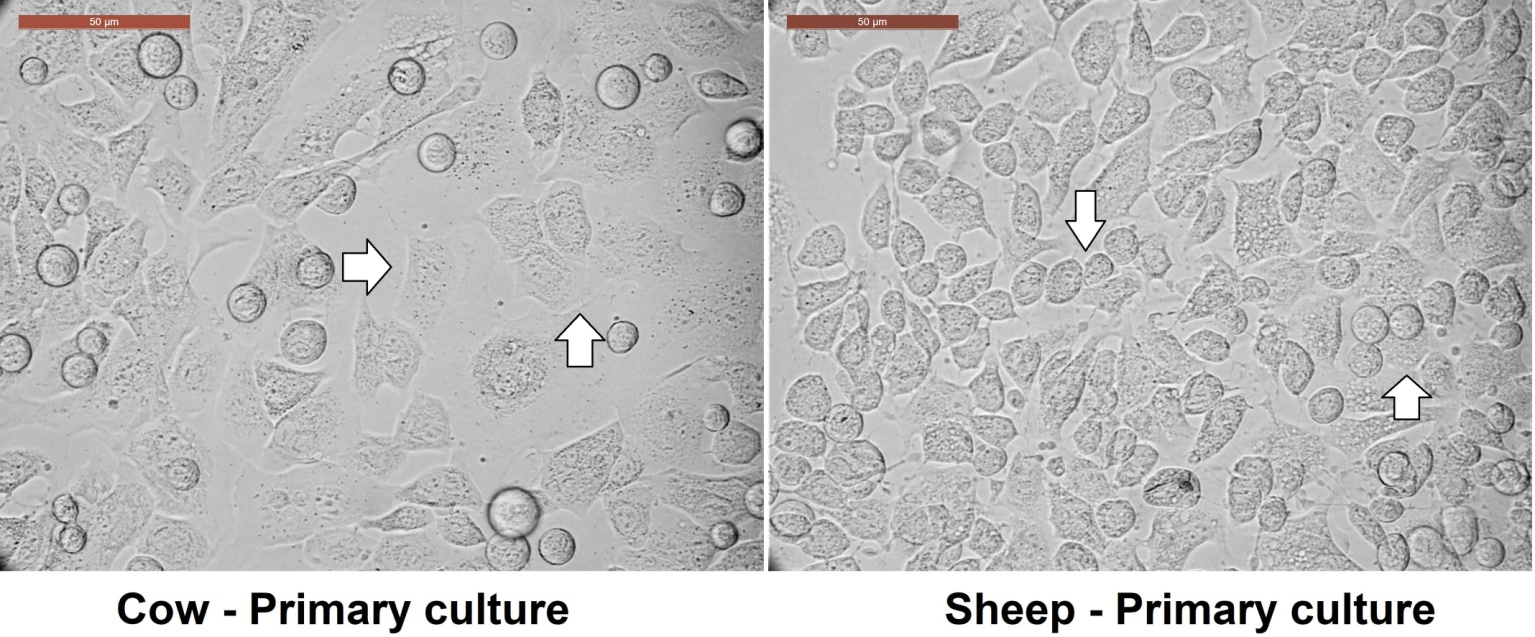
**

**Supplemental Fig. S2.** **High magnification of cow and sheep primary cultured fibroblasts after exposure to acute heat shock (45 °C for 4 h).** Several cow cells remained attached (attached cells are 52.2 ± 3.4%) to the cell culture dish (arrows), while in sheep, the majority of the cells lost their attachment to the culture dish (attached cells are 25.3 ± 5.4%, *P* < 0.05). The numbers and proportions of attached and non-attached cells were reported and analyzed with ImageJ software and data were analyzed by Student’s *t*-test.

**
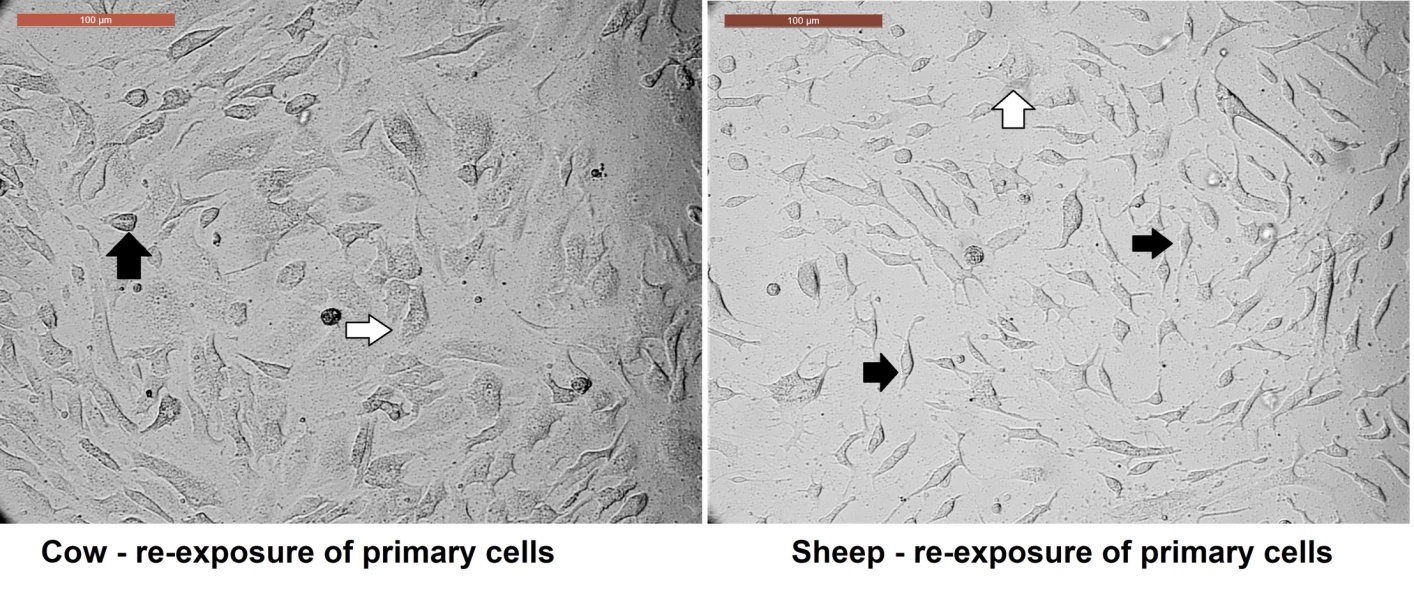
**

**Supplemental Fig. S3.** **High magnification of the recovered cow and sheep primary fibroblasts when re-exposed to acute heat shock (4 °C for 4 h**). In cow cells, several cells remained attached (attached cells are 38.2 ± 3.6%) to the cell culture dish (arrows), while in sheep, the majority of the cells lost their attachment to the culture dish (attached cells are 13.3 ± 4.4%, *P* < 0.05). Numbers and proportions of attached and non-attached cells were reported and analyzed with ImageJ software and the data were analyzed by Student’s *t*-test.


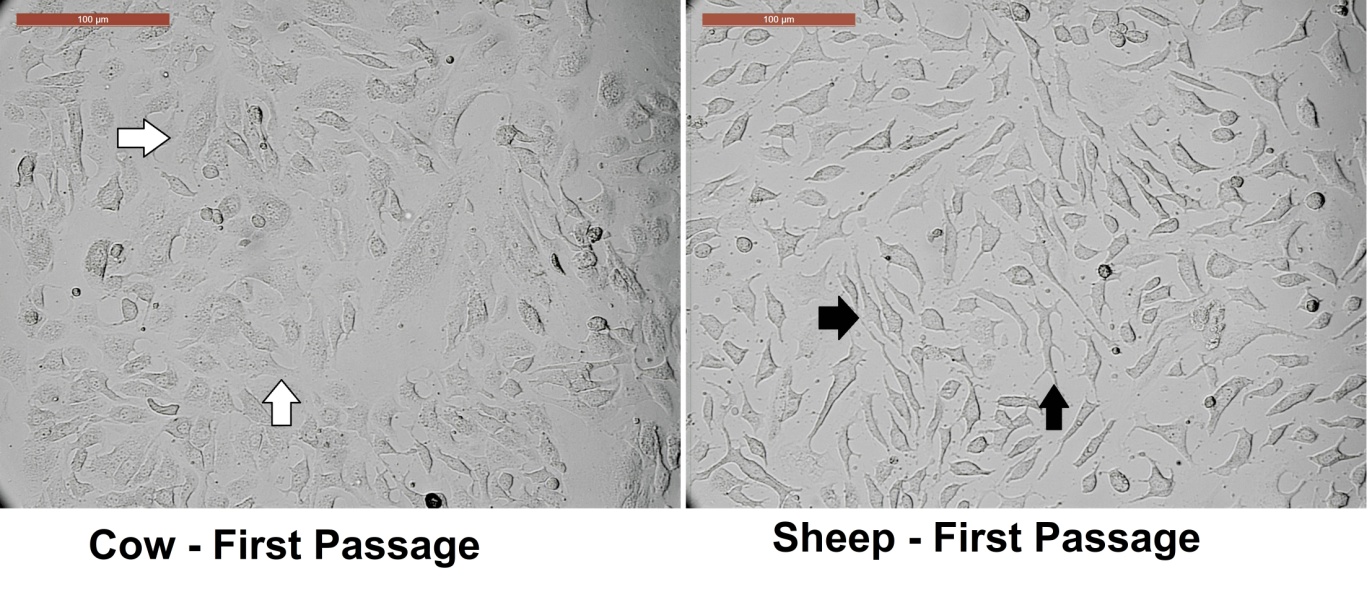


**Supplemental Fig. S4.** **High magnification of the first passage cow and sheep fibroblasts exposed to acute heat shock (4 °C for 4 h).** In cow cells, several cells remained attached (attached cells are 45.1 ± 2.9%) to the cell culture dish (arrows), while the majority of sheep cells lost their spindle architecture (arrows) and attachment to the culture dish (attached cells are 22.1 ± 2.5%, *P* < 0.05). Numbers and proportions of attached and non-attached cells were reported and analyzed with ImageJ software and data were analyzed by Student’s *t*-test.
