# Supplementary material for: Effects of Acute Hyperthermia on the Thermotolerance of Cow and Sheep Skin-Derived Fibroblasts
Source: Animals (Basel). 2020 Mar 25;10(4):545. doi: 10.3390/ani10040545 (PMC7222367; doi:10.3390/ani10040545)
Supplement: Supplementary file 1 [file animals-10-00545-s001.zip › Suppl-Table-1-pimers.docx]

**Supplemental Table S1**: Primers used for relative quantitative PCR.

| **Species** | **Gene name** | Forward (5’---3’) | Reverse (5’---3’) | Size (bp) | Accession number |
| --- | --- | --- | --- | --- | --- |
| **Cow *(Bos taurus)*** | ***GAPDH*** | GGGTCATCATCTCTGCACCT | GGTCATAAGTCCCTCCACGA | 176 | NM_001034034.2 |
|  | ***ACTB*** | ACTTGCGCAGAAAACGAGAT | CACCTTCACCGTTCCAGTTT | 121 | NM_173979.3 |
|  | ***HSP90AA1*** | GACGAGCTCCTTTTGACCTG | CCTCAGAATCCACCACACCT | 144 | NM_001012670.2 |
|  | ***HSP70 (HSPA1A)*** | TGCTGAGGATCATCAACGAG | CCCTCCCAGATCAAAGATGA | 101 | NM_203322.3 |
|  | *TP53* | CCTCTCCACAGCCAAAGAAG | AGAGCATCCTTCAGCTCCAA | 121 | X81704.1 |
|  | *BAX* | TCTGACGGCAACTTCAACTG | TCGAAGGAAGTCCAATGTCC | 135 | NM_173894.1 |
|  | *BCL2* | CATCGTGGCCTTCTTTGAGT | CGGTTCAGGTACTCGGTCAT | 111 | NM_001166486.1 |
|  | *BECN1* | AGGAGCTGCCGTTGTACTGT | CACTGCCTCCTGTGTCTTCA | 189 | NM_001033627.2 |
| **Sheep *(Ovis aries)*** | ***GAPDH*** | GGGTCATCATCTCTGCACCT | GGTCATAAGTCCCTCCACGA | 176 | NM_001190390.1 |
|  | ***ACTB*** | CTCTTCCAGCCTTCCTTCCT | GGGCAGTGATCTCTTTCTGC | 178 | NM_001009784.3 |
|  | ***HSP90AA1*** | GACGAGCTCCTTTTGACCTG | CCTCAGAATCCACCACACCT | 144 | XM_027957416.1 |
|  | ***HSP70 (HSPA1A)*** | CGGAGACAAGCCTAAAGTGC | TCGGCGATCTCTTTCATCTT | 105 | NM_001267874.1 |
|  | *TP53* | GTTCCGAGAGCTGAATGAGG | CAGTCTGAGTCAGGCCCTTC | 162 | NM_001009403.1 |
|  | *BAX* | GACGGCCTCCTCTCCTACTT | CTCAGCCCATCTTCTTCCAG | 106 | XM_027978594.1 |
|  | *BCL2* | CGAGAAGGGGAAAAATCACA | AACCGGAGATCTCAAGAGCA | 108 | XM_012103831.3 |
|  | *BECN1* | AGGAGCTGCCGTTGTACTGT | CACTGCCTCCTGTGTCTTCA | 189 | XM_004012945.4 |
