# Supplementary material for: Effects of Acute Hyperthermia on the Thermotolerance of Cow and Sheep Skin-Derived Fibroblasts
Source: Animals (Basel). 2020 Mar 25;10(4):545. doi: 10.3390/ani10040545 (PMC7222367; doi:10.3390/ani10040545)
Supplement: Supplementary file 1 [file animals-10-00545-s001.zip › Suppl-Tables-2&3-fold-analysis.docx]

**Supplemental Table S2.** Correlation coefficient between fold-changes of different mRNA transcript in primary culture and first passage of fibroblasts in cow.

|  | ***HSP90*** | ***HSP70*** | ***P53*** | ***BAX*** | ***BCL2*** | ***BAX/BCL2*** | ***BECN1*** |
| --- | --- | --- | --- | --- | --- | --- | --- |
| ***HSP90*** | 1 |  |  |  |  |  |  |
| ***HSP70*** | 0.86084 | 1 |  |  |  |  |  |
| ***P53*** | -0.75694 | -0.31906 | 1 |  |  |  |  |
| ***BAX*** | 0.853845 | 0.47014 | -0.98647 | 1 |  |  |  |
| ***BCL2*** | -0.99863 | -0.88627 | 0.721733 | -0.82546 | 1 |  |  |
| ***BAX/BCL2*** | 0.930038 | 0.613621 | -0.94411 | 0.985383 | -0.90955 | 1 |  |
| ***BECN1*** | -0.96491 | -0.96426 | 0.55878 | -0.6872 | 0.977319 | -0.80091 | 1 |

Yellow highlight: strong correlation.

Green highlight: moderate correlation.

“+” positive correlation.

“-“ negative correlation.

**Supplemental Table S3.** Correlation coefficient between fold-changes of different mRNA transcript in primary culture and first passage of fibroblasts in sheep.

|  | ***HSP90*** | ***HSP70*** | ***P53*** | ***BAX*** | ***BCL2*** | ***BAX/BCL2*** | ***BECN1*** |
| --- | --- | --- | --- | --- | --- | --- | --- |
| ***HSP90*** | 1 |  |  |  |  |  |  |
| ***HSP70*** | 0.355285 | 1 |  |  |  |  |  |
| ***P53*** | 0.743731 | -0.36063 | 1 |  |  |  |  |
| ***BAX*** | 0.570539 | -0.56498 | 0.97333 | 1 |  |  |  |
| ***BCL2*** | -0.69481 | 0.42542 | -0.99752 | -0.98707 | 1 |  |  |
| ***BAX/BCL2*** | 0.673975 | -0.4511 | 0.995098 | 0.991246 | -0.99959 | 1 |  |
| ***BECN1*** | 0.147826 | -0.87197 | 0.771077 | 0.896588 | -0.81401 | 0.830269 | 1 |

Yellow highlight: strong correlation.

Green highlight: moderate correlation.

“+” positive correlation.

“-“ negative correlation.
